# Correction: Correlation between novel inflammatory markers and carotid atherosclerosis: A retrospective case-control study

**DOI:** 10.1371/journal.pone.0327051

**Published:** 2025-06-25

**Authors:** Man Liao, Lihua Liu, Lijuan Bai, Ruiyun Wang, Yun Liu, Liting Zhang, Jing Han, Yunqiao Li, Benling Qi

The first two authors, Man Liao and Lihua Liu, should be noted as joint first authors of this work.
