# Supplementary material for: Correlation between novel inflammatory markers and carotid atherosclerosis: A retrospective case-control study
Source: PLoS One. 2024 May 29;19(5):e0303869. doi: 10.1371/journal.pone.0303869 (PMC11135694; doi:10.1371/journal.pone.0303869)
Supplement: Attachment — Submitted filename: Minor Revision.docx [file pone.0303869.s002.docx]

**PLOS ONE**

**Correlation between Novel Inflammatory Markers and Carotid Atherosclerosis: a Cross-Sectional Study**

--Manuscript Draft--

| **Manuscript Number:** | PONE-D-23-42287 |
| --- | --- |
| **Article Type:** | Research Article |
| **Full Title:** | Correlation between Novel Inflammatory Markers and Carotid Atherosclerosis: a Cross-Sectional Study |
| **Short Title:** | Correlation between Novel Inflammatory Markers and Carotid Atherosclerosis |
| **Corresponding Author:** | benling qi  Huazhong University of Science and Technology Tongji Medical College First Clinical College Union Hospital  wuhan, CHINA |
| **Keywords:** | atherosclerosis, carotid atherosclerosis, novel inflammatory marker, neutrophil to lymphocyte platelet ratio, NLPR. |
| **Abstract:** | Objective Carotid atherosclerosis is a chronic inflammatory disease, which is a major cause of ischemic stroke. The purpose of this study was to analyze the relationship between carotid atherosclerosis and novel inflammatory markers, including platelet to lymphocyte ratio (PLR), neutrophil to lymphocyte ratio (NLR), lymphocyte to monocyte ratio (LMR), platelet to neutrophil ratio (PNR), neutrophil to lymphocyte platelet ratio (NLPR), systemic immune-inflammation index (SII), systemic inflammation response index (SIRI), and aggregate index of systemic inflammation (AISI), in order to find the best inflammatory predictor of carotid atherosclerosis. Method We included 10015 patients who underwent routine physical examinations at the physical examination center of our hospital from January 2016 to December 2019, among whom 1910 were diagnosed with carotid atherosclerosis. The relationship between novel inflammatory markers and carotid atherosclerosis was analyzed by logistic regression, and the effectiveness of each factor in predicting carotid atherosclerosis was evaluated by receiver operating characteristic (ROC) curve and area under the curve (AUC). Resulit The level of PLR, LMR and PNR in the carotid atherosclerosis group were lower than those in the non-carotid atherosclerosis group, while NLR, NLPR, SII, SIRI and AISI in the carotid atherosclerosis group were significantly higher than those in the non-carotid atherosclerosis group. Logistic regression analysis showed that PLR, NLR, LMR, PNR, NLPR, SII, SIRI, AISI were all correlated with carotid atherosclerosis. The AUC value of NLPR was the highest, which was 0.67, the cut-off value was 0.78, the sensitivity was 65.8%, and the specificity was 57.3%. The prevalence rate of carotid atherosclerosis was 12.4% below the cut-off, 26.6% higher than the cut-off, and the prevalence rate increased by 114.5%. Conclusion New inflammatory markers were significantly correlated with carotid atherosclerosis, among which NLPR was the optimum inflammatory marker to predict the risk of carotid atherosclerosis. |
| **Order of Authors:** | Benling Qi |
|  | Man Liao |
|  | Yun Liu |
|  | Ruiyun Wang |
|  | Lihua Liu |
|  | Lijuan Bai |
|  | Jing Han |
|  | Liting Zhang |
|  | Yunqiao Li |
| **Additional Information:** |  |
| **Question** | **Response** |

| **Financial Disclosure**  Enter a financial disclosure statement that describes the sources of funding for the work included in this submission. Review the [submission guidelines](http://journals.plos.org/plosone/s/submission-guidelines#loc-financial-disclosure-statement) for detailed requirements. View published research articles from [PLOS ONE](https://journals.plos.org/plosone/) for specific examples.  This statement is required for submission and **will appear in the published article** if the submission is accepted. Please make sure it is accurate. | Yes |
| --- | --- |
| **Funded studies**  Enter a statement with the following details:   - Initials of the authors who received each award - Grant numbers awarded to each author - The full name of each funder - URL of each funder website - Did the sponsors or funders play any role in the study design, data collection and analysis, decision to publish, or preparation of the manuscript? |  |
| Did you receive funding for this work? |  |
| Please add funding details.  as follow-up to "**Financial Disclosure**  Enter a financial disclosure statement that describes the sources of funding for the work included in this submission. Review the [submission guidelines](http://journals.plos.org/plosone/s/submission-guidelines#loc-financial-disclosure-statement) for detailed requirements. View published research articles from [PLOS ONE](https://journals.plos.org/plosone/) for specific examples.  This statement is required for submission and **will appear in the published article** if the submission is accepted. Please make sure it is accurate. | The study was supported by the National Natural Science Foundation of China (Grant No.81571373, No.81601217, No.82001491), Natural Science Foundation of Hubei Province of China (Grant No. 2017CFB627), Health Commission of Hubei Province scientific research project (Grant No. WJ2021M247) and Scientific Research Fund of Wuhan Union Hospital (Grant No.2019). |

| **Funded studies**  Enter a statement with the following details:   - Initials of the authors who received each award - Grant numbers awarded to each author - The full name of each funder - URL of each funder website - Did the sponsors or funders play any role in the study design, data collection and analysis, decision to publish, or preparation of the manuscript? |  |
| --- | --- |
| Did you receive funding for this work?" |  |
| Please select the country of your main research funder (please select carefully as in some cases this is used in fee calculation).  as follow-up to "**Financial Disclosure**  Enter a financial disclosure statement that describes the sources of funding for the work included in this submission. Review the [submission guidelines](http://journals.plos.org/plosone/s/submission-guidelines#loc-financial-disclosure-statement) for detailed requirements. View published research articles from [PLOS ONE](https://journals.plos.org/plosone/) for specific examples.  This statement is required for submission and **will appear in the published article** if the submission is accepted. Please make sure it is accurate. | CHINA - CN |
| **Funded studies**  Enter a statement with the following details:   - Initials of the authors who received each award - Grant numbers awarded to each author - The full name of each funder - URL of each funder website - Did the sponsors or funders play any role in the study design, data collection and analysis, decision to publish, or preparation of the manuscript? |  |
| Did you receive funding for this work?" |  |
| **Competing Interests** | The authors have declared that no competing interests exist. |

| Use the instructions below to enter a competing interest statement for this submission. On behalf of all authors, disclose any [competing interests](http://journals.plos.org/plosone/s/competing-interests) that could be perceived to bias this work—acknowledging all financial support and any other relevant financial or non- financial competing interests.  This statement is required for submission and **will appear in the published article** if the submission is accepted. Please make sure it is accurate and that any funding sources listed in your Funding Information later in the submission form are also declared in your Financial Disclosure statement.  View published research articles from  [PLOS ONE](http://journals.plos.org/plosone/) for specific examples. |  |
| --- | --- |
| **NO authors have competing interests**  Enter: The authors have declared that no competing interests exist.  **Authors with competing interests**  Enter competing interest details beginning with this statement:  I have read the journal's policy and the authors of this manuscript have the following competing interests: [insert competing interests here] |  |
| * typeset |  |
| **Ethics Statement**  Enter an ethics statement for this submission. This statement is required if the study involved:   - Human participants - Human specimens or tissue - Vertebrate animals or cephalopods - Vertebrate embryos or tissues | This is a retrospective case-control study. All methods were carried out in accordance with relevant guidelines and regulations and no important aspects of the study have been omitted. Patients’ personal information is being kept confidentially. The study complies with the Declaration of Helsinki and the ethical approval of the study was obtained from the Clinical Trial Ethics Committee of Huazhong University of Science and Technology at Sep. 4th 2023 ([2023]ID:0611). The ethics committee exempted the need for informed consent. |

| - Field research   Write "N/A" if the submission does not require an ethics statement.  General guidance is provided below. Consult the [submission guidelines](http://journals.plos.org/plosone/s/submission-guidelines#loc-guidelines-for-specific-study-types) for detailed instructions. **Make sure that all information entered here is included in the Methods section of the manuscript.** |  |
| --- | --- |

| **Format for specific study types**  **Human Subject Research (involving human participants and/or tissue)**   - Give the name of the institutional review board or ethics committee that approved the study - Include the approval number and/or a statement indicating approval of this research - Indicate the form of consent obtained (written/oral) or the reason that consent was not obtained (e.g. the data were analyzed anonymously)   **Animal Research (involving vertebrate animals, embryos or tissues)**   - Provide the name of the Institutional Animal   Care and Use Committee (IACUC) or other relevant ethics board that reviewed the study protocol, and indicate whether they approved this research or granted a formal waiver of ethical approval   - Include an approval number if one was obtained - If the study involved non-human primates, add additional details about animal welfare and steps taken to ameliorate suffering - If anesthesia, euthanasia, or any kind of animal sacrifice is part of the study, include briefly which substances and/or methods were applied   **Field Research**  Include the following details if this study involves the collection of plant, animal, or other materials from a natural setting:   - Field permit number - Name of the institution or relevant body that granted permission |  |
| --- | --- |
|  |  |
| **Data Availability**  Authors are required to make all data underlying the findings described fully available, without restriction, and from the time of publication. PLOS allows rare exceptions to address legal and ethical concerns. See the [PLOS Data Policy](http://journals.plos.org/plosone/s/data-availability) and [FAQ](http://journals.plos.org/plosone/s/data-availability#loc-faqs-for-data-policy) for detailed information. | No - some restrictions will apply |

| A Data Availability Statement describing where the data can be found is required at submission. Your answers to this question constitute the Data Availability Statement and **will be published in the article**, if accepted. |  |
| --- | --- |
| **Important:** Stating ‘data available on request from the author’ is not sufficient. If your data are only available upon request, select ‘No’ for the first question and explain your exceptional situation in the text box. |  |
| Do the authors confirm that all data underlying the findings described in their manuscript are fully available without restriction? |  |
| **Describe where the data may be found in full sentences. If you are copying our sample text, replace any instances of XXX with the appropriate details.**   - If the data are **held or will be held in a public repository**, include URLs, accession numbers or DOIs. If this information will only be available after acceptance, indicate this by ticking the box below. For example: All XXX files are available from the XXX database (accession number(s) XXX, XXX.). - If the data are all contained **within the**   **manuscript and/or Supporting Information files**, enter the following: All relevant data are within the manuscript and its Supporting Information files.   - If neither of these applies but you are able to provide **details of access elsewhere**, with or without limitations, please do so. For example:   Data cannot be shared publicly because of [XXX]. Data are available from the XXX Institutional Data Access / Ethics Committee (contact via XXX) for researchers who meet the criteria for access to confidential data.  The data underlying the results presented in the study are available from (include the name of the third party | Data cannot be shared publicly because of the data involved patient privacy. Data are available from the Wuhan union hospital Institutional Data Access / Ethics Committee (contact via Dr. QI BL for researchers who meet the criteria for access to confidential data. |

| and contact information or URL).   - This text is appropriate if the data are owned by a third party and authors do not have permission to share the data.   * typeset |  |
| --- | --- |
| Additional data availability information: |  |

Manuscript	[Click here to access/download;Manuscript;manuscript.docx](https://www.editorialmanager.com/pone/download.aspx?id=35221698&guid=7ba80df2-13ca-4daa-8c61-3f4ce728a6fb&scheme=1)

1 Title page
2 The title: Correlation between Novel Inflammatory Markers and Carotid
3 Atherosclerosis: a Cross-Sectional Study
4 Running title: Correlation between Novel Inflammatory Markers and Carotid.
5 Atherosclerosis
6 The name(s) of authors: Man Liao#,^1^ MD, Lihua Liu#,^1^ MD, PhD, Lijuan Bai^1^ MD,
7 PhD, Ruiyun Wang^1^ MD, PhD, Yun Liu^1^ MD, PhD, Liting Zhang,^1^ MD, Jing Han,^1^
8 MD, Yunqiao Li*,^1^ MD, PhD, Benling Qi*,^1^ MD, PhD.
9 Man Liao† and Lihua Liu† contribute equally to this work and share first authorship.
10 Yunqiao Li† and Benling Qi† are both the corresponding authors, and Benling Qi
11 takes primary responsibility for the entire paper.
12 The institution(s) where the work was performed:
13 ^1^Department of Geriatrics, Union Hospital, Tongji Medical College, Huazhong
14 University of Science and Technology, 1277 Jiefang Avenue, Jianghan District,
15 430022, Wuhan, Hubei Province, China.
16 *Correspondent Author:
17 Yunqiao Li, M.D, Ph.D.
18 Address: Department of general, Union Hospital, Tongji Medical College, Huazhong
19 University of Science and Technology, 1277 Jiefang Avenue, Wuhan, Hubei, 430022,
20 China.
21 E-mail: liyunqiao @hust.edu.cn
22 Benling Qi, M.D, Ph.D.
23 Address: Department of general, Union Hospital, Tongji Medical College, Huazhong
24 University of Science and Technology, 1277 Jiefang Avenue, Wuhan, Hubei, 430022,
25 China.
26 Tel: +86 13886024487
27 E-mail: [qibenlingok_2015@163.com](mailto:qibenlingok_2015@163.com)
28 The number of figures: 3
29 The number of tables: 3

30

## 31 Abstract

32 **Objective:** Carotid atherosclerosis is a chronic inflammatory disease, which is a
33 major cause of ischemic stroke. The purpose of this study was to analyze the
34 relationship between carotid atherosclerosis and novel inflammatory markers,
35 including platelet to lymphocyte ratio (PLR), neutrophil to lymphocyte ratio
36 (NLR), lymphocyte to monocyte ratio (LMR), platelet to neutrophil ratio
37 (PNR),	neutrophil	to	lymphocyte	platelet	ratio	(NLPR),	systemic
38 immune-inflammation index (SII), systemic inflammation response index
39 (SIRI), and aggregate index of systemic inflammation (AISI), in order to find
40 the best inflammatory predictor of carotid atherosclerosis.
41 **Method:** We included 10015 patients who underwent routine physical
42 examinations at the physical examination center of our hospital from January
43 2016 to December 2019, among whom 1910 were diagnosed with carotid
44 atherosclerosis. The relationship between novel inflammatory markers and
45 carotid	atherosclerosis	was	analyzed	by	logistic	regression,	and	the
46 effectiveness of each factor in predicting carotid atherosclerosis was evaluated
47 by receiver operating characteristic (ROC) curve and area under the curve
48 (AUC).
49 **Resulit:** The level of PLR, LMR and PNR in the carotid atherosclerosis group
50 were lower than those in the non-carotid atherosclerosis group, while NLR,
51 NLPR, SII, SIRI and AISI in the carotid atherosclerosis group were
52 significantly higher than those in the non-carotid atherosclerosis group.
53 Logistic regression analysis showed that PLR, NLR, LMR, PNR, NLPR, SII,
54 SIRI, AISI were all correlated with carotid atherosclerosis. The AUC value of
55 NLPR was the highest, which was 0.67, the cut-off value was 0.78, the
56 sensitivity was 65.8%, and the specificity was 57.3%. The prevalence rate of
57 carotid atherosclerosis was 12.4% below the cut-off, 26.6% higher than the
58 cut-off, and the prevalence rate increased by 114.5%.
59 **Conclusion:** New inflammatory markers were significantly correlated with
60 carotid atherosclerosis, among which NLPR was the optimum inflammatory
61 marker to predict the risk of carotid atherosclerosis.
62 **Key word:** atherosclerosis, carotid atherosclerosis, novel inflammatory marker,
63 neutrophil to lymphocyte platelet ratio, NLPR.

64

## 65 Introduction

66 Cardiovascular was always the main cause of premature death and rising
67 healthcare costs [1, 2]. From 1990 to 2019, the total number cardiovascular
68 disease cases has nearly doubled globally, while the number of cases and
69 deaths from peripheral artery disease tripled [3]. Atherosclerosis, a major
70 pathological process in most cardiovascular diseases, which may occuar as
71 early as childhood and remain latent in the body for a long time [4]. Early
72 detection of arterial disease in seemingly healthy individuals focuses on the
73 peripheral arteries, especially the carotid arteries [5]. Carotid atherosclerotic
74 plaque is an independent risk factor for stroke and coronary heart disease [6].
75 Atherosclerosis is widely recognized as a chronic inflammatory disease of the
76 blood vessels caused by the accumulation of low density lipoprotein cholesterol
77 [7]. Chronic inflammation is associated with endothelial dysfunction,
78 leukocyte recruitment, transformation of monocytes into macrophages and
79 eventually into foam cells, smooth muscle cell migration and other processes [7].
80 Chronic inflammation is involved in the whole process of the occurrence and
81 development of atherosclerosis and is the core of atherosclerosis.
82 In clinical practice, peripheral blood cell count is often used as a predictor
83 and evaluation factor for the severity of inflammation and treatment outcome
84 of acute inflammatory diseases, such as lung infection and sepsis. Platelet to
85 lymphocyte ratio (PLR), neutrophil to lymphocyte ratio (NLR), lymphocyte to
86 monocyte ratio (LMR), platelet to neutrophil ratio (PNR), neutrophil to
87 lymphocyte platelet ratio (NLPR), systemic immune-inflammation index (SII),
88 systemic inflammation response index (SIRI), and aggregate index of systemic
89 inflammation (AISI) are commonly used blood cell count derived values in
90 clinical practice, also known as novel inflammatory markers. These novel
91 inflammatory markers have repeatedly demonstrated their potential value in
92 the early prediction and prognosis of cardiovascular disease [8-13]. However,
93 the relationship between these readily available inflammation markers and
94 atherosclerosis, especially carotid atherosclerosis (CAS), has not yet been
95 clear, which nedds further research to be confirmed.
96 Therefore, this study aimed to explore the correlation between novel
97 inflammatory markers and CAS, in order to find a better early warning
98 indicator of clinical carotid atherosclerosis.

## 99 Method

100 **Study design**
101 This is a retrospective case-control study. All methods were carried out in
102 accordance with relevant guidelines and regulations and no important aspects of the
103 study have been omitted. Patients’ personal information is being kept confidentially.
104 The study complies with the Declaration of Helsinki and the ethical approval of the
105 study was obtained from the Ethics Committee of Union Hospital, Tongji Medical
106 College, Huazhong University of Science and Technology at Sep. 4th 2023
107 ([2023]ID:0611). The ethics committee exempted the need for informed consent.

## 108 Population

109 In this study, we included 14,115 patients who underwent routine physical
110 examinations at our physical examination center from January 2016 to
111 December 2019. The first time we accessed the data was on September 10,
112 2023. The exclusion criteria of participants were as follows: (1) Age <18 years;
113 (2) Receiving or being receiving anti-inflammatory therapy within 6 months;
114 (3) Critically ill patients with unstable vital signs; (4) Have received or are
115 receiving glucocorticoid therapy within 6 months; (5) Cervical vascular
116 ultrasound examination was not performed; (6) Incomplete clinical data or
117 incomplete personal information. After screening and exclusion, a total of
118 10015 patients were included and divided into CAS group and non-CAS group
119 according to diagnosis.

## 120 Collected clinical data and laboratory indicators and definition of

121 **inflammatory markers**
122 Age, gender, height, and weight of the patient at admission were obtained
123 from the hospital electronic system, and BMI was calculated accordin g to
124 BMI= weight/height ^2. Proper amount of venous blood was extracted by
125 professional nurses with nursing qualification during the fasting period and
126 sent to the laboratory for analysis to obtain white blood cell count (WBC),
127 neutrophil count (NC), lymphocyte count (LC), monocyte count (MC), platelet
128 (PLT), neutrophil percentage (NP ), lymphocyte percentage (LP), monocyte
129 percentage (MP), PLR, NLR, LMR, PNR, NLPR, SII, SIRI, AISI and
130 biochemical index, such as aspartate aminotransferase (AST), triglyceride (TG),
131 total cholesterol (TC), high density lipoprotein cholesterol (HDL-C), low densit

lipoprotein cholesterol (LDL-C), uric acid (UA), urea nitrogen (BUN), creatinine.

132 The estimated glomerular filtration rate (eGFR) was calculated based on the
133 modified MDRD equation. SIRI was defined as neutrophil count*monocyte
134 count/lymphocyte count. SII was calculated using the formula: NLR*platele.
135 AISI	was	calculated	using	the	formula:	(neutrophil	count*monocyte
136 count*platelet)/lymphocyte count. NLPR was calculated using the formula:
137 neutrophil	count*100/lymphocyte	count*platelet.	Comorbidity,	smoking
138 history and drinking history were obtained from the patient's personal history.

## 139 Diagnostic criteria for carotid atherosclerosis

140 Carotid atherosclerosis is diagnosed in one of the following situations: (1) A
141 clear history of atherosclerosis or revascularization treatment; (2) Carotid
142 artery ultrasound showed atherosclerotic plaque or carotid intima-media
143 thickness of 1.0mm or more[5].

## 144 Statistical analysis

145 Shapiro-Wilkstest is used to test whether the continuous variables obey
146 normal distribution, and the continuous variables with normal distribution are
147 represented by mean ± standard deviation (SD). Continuous variables that are not
148 normally distributed are expressed as medians with interquartile range (IQR). The
149 categorical variable is represented by number with percentages (%). ANOVA
150 tests	(conforming	to	normal	distribution)	and	Kruskal-Wallis	tests
151 (non-conforming to normal distribution) were used for the differences between
152 of continuous data among CAS and non-CAS. Chi-square test was used to compare
153 the difference of Categorical data between patients with CAS and non-CAS. Logistic
154 regression analysis was used to evaluate the correlation between various
155 inflammatory indicators and CAS after adjusting factors such as age, gender,
156 BMI, serum biochemical indicators and clinical diagnosis and other factors. In
157 Logistic regression analysis, forward selection method was used to identify
158 CAS risk factors. Finally, the efficiency of each inflammatory markers to CAS
159 was evaluated by receiver operating characteristic (ROC) curve. P<0.05 was
160 considered to be statistically significant. All statistical analyses and diagrams were
161 done using SPSS (version 23.0) or R language.

## 162 Result

163 **Clinical baseline data**
164 A total of 10015 patients aged 18-94 were enrolled in the study, including
165 1910 (19.1%) in the CAS group. Table 1 shows the baseline characteristics
166 between the two groups. There were no significant differences in BMI, PLR
167 and dyshepatia between the two groups (P>0.05). While age, gender, WBC,
168 NC, LC, MC, PLT, NP, LP, MP, NLR, LMR, PNR, NLPR, SII, SIRI, AISI,
169 AST, TG, TC, HDL_C, LDL_C, BUN, creatinine, eGFR, UA, smoking history,
170 drinking history, renaldysfuncyion, hyperuricemia, fatty liver, dyslipidemia,
171 hypertension, diabetes and osteoporosis were significantly different between
172 the two groups(P<0.05).
173 Table 1. Baseline characteristics between CAS and Non-CAS

|  | Non-CAS | CAS | P value |
| --- | --- | --- | --- |
| N(%) | 8105(80.9) | 1910(19.1) |  |

| Age,year | 43.21 ± 12.65 | 66.12 ± 12.07 | 0.000 |
| --- | --- | --- | --- |
| Male, N(%) | 5105(63.0) | 1605(84.0) | 0.000 |
| BMI, Kg/m2 | 23.80 ± 3.30 | 24.53 ± 3.19 | 0.148 |
| WBC | 6.11 ± 1.46 | 6.29 ± 1.59 | 0.000 |
| NC | 3.50 ± 1.09 | 3.78 ± 1.25 | 0.000 |
| LC | 2.08 ± 0.55 | 1.91 ± 0.56 | 0.000 |
| MC | 0.36 ± 0.12 | 0.39 ± 0.13 | 0.000 |
| PLT | 235.57 ± 54.26 | 212.40 ± 52.94 | 0.000 |
| NP | 56.69 ± 7.44 | 59.51 ± 7.82 | 0.000 |
| LP | 34.48 ± 7.05 | 31.04 ± 7.44 | 0.000 |
| MP | 5.95 ± 1.53 | 6.35 ± 1.67 | 0.000 |
| PLR | 120.07 ± 38.23 | 118.90 ± 41.34 | 0.239 |
| NLR | 1.76 ± 0.61 | 2.12 ± 0.91 | 0.000 |
| LMR | 6.19 ± 2.10 | 5.24 ± 1.93 | 0.000 |
| PNR | 72.54 ± 24.68 | 60.64 ± 20.90 | 0.000 |
| NLPR | 0.78 ± 0.31 | 1.06 ± 0.54 | 0.000 |
| SII | 418.42 ± 188.35 | 452.74 ± 237.00 | 0.000 |
| SIRI | 0.65 ± 0.36 | 0.86 ± 0.56 | 0.000 |
| AISI | 156.07 ± 104.87 | 186.33 ± 137.93 | 0.000 |
| TP, g/L | 74.86 ± 4.12 | 74.35 ± 4.40 | 0.000 |
| Albumin, g/L | 47.27 ± 2.54 | 46.30 ± 2.68 | 0.000 |
| Globulin, g/L | 27.58 ± 3.31 | 28.05 ± 3.77 | 0.000 |
| AGR | 1.74 ± 0.23 | 1.68 ± 0.25 | 0.000 |
| AST, U/L | 25.80 ± 19.48 | 24.48 ± 18.33 | 0.008 |
| TG, mmol/L | 1.58 ± 1.23 | 1.65 ± 1.09 | 0.016 |
| TC, mmol/L | 4.75 ± 0.86 | 4.84 ± 1.01 | 0.000 |
| HDL_C, mmol/L | 1.40 ± 0.34 | 1.37 ± 0.33 | 0.000 |
| LDL_C, mmol/L | 2.74 ± 0.70 | 2.84 ± 0.83 | 0.000 |
| BUN, mmol/L | 4.76 ± 1.21 | 5.65 ± 1.54 | 0.000 |
| Creatinine, umol/L | 71.46 ± 14.61 | 79.76 ± 19.64 | 0.000 |
| eGFR, mL/min/1.73m2 | 109.06 ± 22.75 | 93.81 ± 22.49 | 0.000 |
| Uric acid, mmol/L | 363.82 ± 96.91 | 375.34 ± 88.49 | 0.000 |
| Smoking history,N(%) | 2642(32.6) | 830(43.5) | 0.000 |
| Drinking history,N(%) | 1901(23.5) | 591(30.9) | 0.000 |
| Renaldysfuncyion,N(%) | 31(0.4) | 103(5.4) | 0.000 |
| Dyshepatia,N(%) | 83(1.0) | 13(0.7) | 0.166 |
| Dyslipidemia,N(%) | 5231(64.5) | 1402(73.4) | 0.000 |
| Hyperuricemia,N(%) | 4044(49.9) | 1096(57.4) | 0.000 |
| Fatty liver,N(%) | 2484(30.6) | 718(37.6) | 0.000 |
| Diabetes,N(%) | 339(4.2) | 297(15.5) | 0.000 |
| Osteoporosis,N(%) | 745(9.2) | 446(23.4) | 0.000 |
| Hypertension,N(%) | 1581(19.5) | 835(43.7) | 0.000 |

174 Continuous variables are presented as mean ± S.D. Categorical data are presented as number
175 (percentages).
176 CAS, carotid atherosclerosis; BMI, body mass index; WBC, white blood cell; NC, neutrophil count;
177 LC, lymphocyte count; MC, monocyte count; PLT, platelet; NP, neutrophil percentage; LP, lymphocyte
178 percentage; MP, monocyte percentage ; PLR, platelet to lymphocyte ratio; NLR, neutrophil to
179 lymphocyte ratio; LMR , lymphocyte to monocyte ratio; PNR, platelet to neutrophil ratio; NLPR,
180 neutrophil to lymphocyte platelet ratio; SII, systemic immune-inflammation index; SIRI, systemic
181 inflammation response index; AISI, aggregate index of systemic inflammation; TP, total protein; AGR,
182 albumin-globulin ratio; AST, aspartate aminotransferase; TG, triglyceride; TC, total cholesterol;
183 HDL_C, high density lipoprotein cholesterol; LDL_C, low density lipoprotein cholesterol; BUN, blood
184 urea nitrogen; eGFR, estimated glomerular filtration rate.

## 185 Relationship between novel inflammatory markers and CAS

186 The relationship between novel inflammation markers and CAS was
187 observed by spline smoothing plot, shown in figure 1. LMR, PNR were
188 negatively correlated with CAS, while PLR, NLR, NLPR, SII, SIRI, AISI,
189 NLPR were positively correlated with CAS.

191

192 Fig 1.	Spline smoothing plot	between novel	inflammation markers and carotid
193 atherosclerosis. A non- linear relationship between novel inflammation markers and carotid
194 atherosclerosis after adjusting for age, gender, body mass index, aspartate aminotransferase,
195 triglyceride, total cholesterol, high density lipoprotein cholesterol, low density lipoprotein cholesterol,
196 estimated glomerular filtration rate, uric acid, hypertension, diabetes, osteoporosis, fatty liver, smoking
197 history, drinking history. CAS, carotid atherosclerosis; PLR, platelet to lymphocyte ratio; NLR,
198 neutrophil to lymphocyte ratio; LMR , lymphocyte to monocyte ratio; PNR, platelet to neutrophil ratio;
199 NLPR, neutrophil to lymphocyte platelet ratio; SII, systemic immune-inflammation index; SIRI,
200 systemic inflammation response index; AISI, aggregate index of systemic inflammation.

## 201 Logistic regression analysis of CAS

202 Univariate	logistic	regression	analysis	of	the	relationship	between
203 inflammatory markers and CAS showed the same difference as that between
204 the two groups. Furthermore, after adjusting for many possible confounders
205 such as age, sex, BMI, all inflammatory markers were statistically significant
206 (P<0.05) with CAS. These infalmmatory markers have varying effects on CAS,
207 and the risk value of NLPR is much higher than other markers (OR=2.35).
208 Their CAS risk value and forest plot were shown in figure 2.

209

210

Fig 2. The ORs and 95% CI of novel inflammatory indicators to CAS risk by logistic regression.

211 Model 1 adjusted for none. Model 2 adjusted for age, sex, and body mass index. Model 3 adjusted for
212 age, gender, body mass index, aspartate aminotransferase, triglyceride, total cholesterol, high density
213 lipoprotein cholesterol, low density lipoprotein cholesterol, estimated glomerular filtration rate, uric
214 acid, hypertension, diabetes, osteoporosis, smoking history, drinking history. Forest plot of novel
215 inflammatory markers for carotid atherosclerosis risk after adjusting for Model 3. PLR,
216 platelet to lymphocyte ratio; NLR, neutrophil to lymphocyte ratio; LMR , lymphocyte to monocyte
217 ratio; PNR, platelet to neutrophil ratio; NLPR, neutrophil to lymphocyte platelet ratio; SII, systemic
218 immune-inflammation index; SIRI, systemic inflammation response index; AISI, aggregate index of
219 systemic inflammation.
220 The efficacy of inflammatory markers to predict CAS was assessed by ROC
221 curve. After adjusting for confounding factors, there were still statistical
222 differences in all inflammatory markers. The ROC curve to evaluate the
223 effectiveness of the above markers in CAS diagnosis is shown in table 2 and
224 figure 3. The AUC values of NLR, SII, SIRI, AISI and NLPR were all in the
225 range of 0.5-0.7. The highest AUC value for NLPR recognition of CAS is 0.67. And
226 NLPR uses the adjusted predictive value of Model 3 to identify the AUC value
227 of CAS as 0.91. The cut-off value calculated by Youden index was that NLPR was
228 0.78, that the sensitivity was 65.8%, and that the specificity was 57.3%. The
229 prevalence rate of carotid atherosclerosis was 12.4% below the cut-off, 26.6%
230 higher than the cut-off, and the prevalence rate increased by 114.5%.
231 Table 2. Evaluation of the predictive effect of the novel inflammatory markers on risk of CAS by ROC
232 curves

|  | AUC (95%CI) | Specificity | Sensitivity | Cut-off | Youden’s index |
| --- | --- | --- | --- | --- | --- |
| PLR | 0.52 (0.50,0.53)^a^ | 0.76 | 0.28 | 92.41 | 0.04 |
| NLR | 0.62(0.60,0.63)^a^ | 0.69 | 0.47 | 1.96 | 0.16 |
| LMR | 0.64(0.62,0.65)^a^ | 0.68 | 0.51 | 5.02 | 0.19 |
| PNR | 0.65(0.63,0.66)^a^ | 0.64 | 0.57 | 61.24 | 0.21 |
| SII | 0.53(0.52,0.55)^a^ | 0.84 | 0.23 | 571.56 | 0.07 |
| SIRI | 0.63(0.62,0.64)^a^ | 0.69 | 0.50 | 0.72 | 0.19 |
| AISI | 0.57(0.55,0.58)^a^ | 0.66 | 0.45 | 163.70 | 0.11 |
| NLPR | 0.67(0.65,0.68)^a^ | 0.57 | 0.66 | 0.78 | 0.23 |
| Model 3-NLPR | 0.91(0.90,0.91)^a^ | 0.80 | 0.85 | 0.18 | 0.65 |

233 CAS, carotid atherosclerosis; ROC, receiver operating curve; AUC, area under the curve; PLR,
234 platelet to lymphocyte ratio; NLR, neutrophil to lymphocyte ratio; LMR , lymphocyte to monocyte
235 ratio; PNR, platelet to neutrophil ratio; NLPR, neutrophil to lymphocyte platelet ratio; SII, systemic
236 immune-inflammation index; SIRI, systemic inflammation response index; AISI, aggregate index of
237 systemic inflammation.
238 ^a^P<0.05.

239

240

Fig 3. Receiver operating characteristic (ROC) curves of all inflammatory markers for identifying

241 carotid atherosclerosis risk. PLR, platelet to lymphocyte ratio; NLR, neutrophil to lymphocyte ratio;
242 LMR , lymphocyte to monocyte ratio; PNR, platelet to neutrophil ratio; NLPR, neutrophil to
243 lymphocyte platelet ratio; SII, systemic immune-inflammation index; SIRI, systemic inflammation
244 response index; AISI, aggregate index of systemic inflammation.

## 245 Subgroup analyses between NLPR and CAS

246 In order to study the CAS risk and interaction of inflammatory markers in
247 people with different clinicopathological characteristics. We further analyzed
248 six subgroups of NLPR and CAS risk indicators (hyperuricemia, fatty liver,
249 dyslipidemia, hypertension, diabetes, osteoporosis). The results showed that
250 NLPR had the highest OR value in population without lipid metabolism
251 abnormalities (Table 3). CAS were significantly associated with NLPR in all
252 subgroups. The interaction among subgroups revealed that NLPR had an
253 interaction	with	hyperuricemia,	fatty	liver,	dyslipidemia,	hypertension,
254 diabetes and osteoporosis, and all of these disease subgroups significantly
255 weakened the risk of NLPR for CAS.
256 Table 3. Association between NLPR and CAS in subgroups

|  | N(%) | OR(95%CI) | P interaction |
| --- | --- | --- | --- |
| Hyperuricemia |  |  | 0.000 |
| No | 4875 | 2.45(1.89,3.17) |  |
| Yes | 5140 | 2.29(1.81,2.90) |  |
| Fatty liver |  |  | 0.000 |
| No | 6813 | 2.59(2.07,3.25) |  |
| Yes | 3202 | 1.98(1.49,2.63) |  |
| Dyslipidemia |  |  | 0.000 |
| No | 3382 | 2.72(1.89,3.87) |  |
| Yes | 6633 | 2.24(1.83,2.74) |  |
| Hypertension |  |  | 0.000 |
| No | 7599 | 2.56(2.04,3.22) |  |
| Yes | 2416 | 2.14(1.64,2.81) |  |
| Diabetes |  |  | 0.003 |
| No | 9379 | 2.38(1.98,2.87) |  |
| Yes | 636 | 2.26(1.37,3.73) |  |
| Osteoporosis |  |  | 0.000 |
| No | 7755 | 2.55(2.08,3.13) |  |
| Yes | 1191 | 1.91(1.31,2.76) |  |

257 Above subgroups were adjusted for age, gender, body mass index, aspartate aminotransferase,
258 triglyceride, total cholesterol, high density lipoprotein cholesterol, low density lipoprotein cholesterol,
259 estimated glomerular filtration rate, uric acid, hypertension, diabetes, osteoporosis, smoking history,
260 drinking history.
261 CAS, carotid atherosclerosis; NLPR, neutrophil to lymphocyte platelet ratio.

262

## 263 Discussion

264 Novel inflammatory markers have been proven to be closely related to
265 various diseases, and their easy availability has important clinical value. In this
266 cross-sectional study, we analyzed the effect of multiple inflammatory
267 markers on CAS, where NLR, SII, SIRI, AISI, NLPR remained significant
268 after adjusting for multiple confounding factors. At the same time, this study
269 further evaluated the value of various inflammatory markers in predicting
270 CAS, among which NLPR had the largest AUC value in identifying CAS risk.
271 Compared with other inflammatory markers, NLPR has a higher value for
272 predicting CAS which may be the best inflammatory indicator for identifying
273 atherosclerosis.
274 Chronic inflammation leads to atherosclerosis and cardiovascular disease.
275 Neutrophils are the most numerous type of white blood cell and play a major
276 role in inflammation. Although neutrophil count is primarily used as a
277 biomarker for acute infection and inflammation, it has also been shown to
278 accelerate chronic inflammation [14]. Monocyte-derived macrophages are
279 important mediators in the atherosclerotic cascade and are associated with
280 plaque	formation	through	infiltration	into	the	subendothelial	layer.
281 Subsequently, uptake of LDL-C complexes leads to the formation of foam cells.
282 In addition to plaque formation, macrophages are involved in extracellular
283 matrix remodeling by secreting pro-inflammatory cytokines and chemokines
284 [15]. In our study, the number of neutrophils and monocytes increased in
285 patients with CAS compared with non-CAS patients, indicating that the higher
286 the number of neutrophils and monocytes, the higher the incidence of CAS. In
287 contrast, lymphocytes slow the progression of atherosclerosis [13]. Our
288 findings were the same, with a reduced number of lymphocytes in patients
289 with CAS, suggesting that the higher the number of lymphocytes, the lower
290 the incidence of CAS. Platelets play two main roles in atherosclerosis:
291 platelets directly adhere to the blood vessel wall to promote plaque formation,
292 and platelets then release inflammatory mediators and chemokines to promote
293 leukocyte recruitment [16]. In our results, platelet counts were reduced in
294 patients with CAS compared to non-CAS patients. The reason for this opposite
295 situation may be that platelets have multiple roles in the formation of
296 atherosclerosis, and in our study, the number of platelets alone was not
297 significantly associated with the development of carotid atherosclerosis. In
298 subsequent binary logistic regression analysis, there was no statistical
299 difference	between	platelet	count	and	CAS	risk	after	adjusting	for
300 confounders.
301 In previous studies, we found that NLR, MLR, and PLR increase the risk of
302 arterial stiffness [17]. In recent years, LMR, PNR, NLPR, SII, SIRI, AISI and
303 other indicators have also proved their clinical significance in cardiovascular
304 diseases.	A small	sample	observational	study	showed	that	the	novel
305 inflammatory markers SIRI, NLR, and LMR were associated with CAS risk in
306 middle-aged and older men [8]. PNR have also been shown to predict
307 mortality in patients with ischemic stroke [9]. SII was also found to be a
308 strong independent predictor of adverse outcomes in patients with acute
309 coronary syndromes [11]. To find the best predictors of CAS, we included
310 these markers in a larger population study at the same time. The results
311 showed that the NLPR had the highest accuracy in identifying the risk of CAS.
312 NLPR is the ratio of neutrophil to lymphocyte*platelet. Initially, NLPR was
313 found to have predictive value in tumor prognosis [18, 19]. Subsequently,
314 NLPR was also found to be associated with the prognosis of acute kidney
315 injury, suppurative liver abscess, severe trauma, and COVID-19 [20-24]. The
316 value of NLPR in cardiovascular disease is equally outstanding. Studies have
317 shown that NLPR is an independent predictor of in-hospital mortality after
318 acute type A aortic dissection [10]. A prospective cohort study found that
319 NLPR was an independent predictor of adverse outcomes in patients with
320 acute coronary syndrome, and patients with a higher NLRP had a higher
321 incidence of major adverse cardiovascular events [25]. In this study, our
322 results directly show that there is a significant correlation between NLPR and
323 CAS, and the higher the level of NLPR, the higher the risk of CAS.
324 In this study, we evaluated the value of multiple inflammatory markers for
325 the risk of CAS and found the best inflammatory marker that can be easily
326 obtained to effectively identify the risk of CAS. In addition, the relatively
327 large sample size ensures the accuracy and reliability of the results.
328 Nevertheless, there are some limitations to this study. First, due to the
329 cross-sectional	design	of	the	study,	a	causal	relationship	between
330 inflammatory markers and CAS cannot be demonstrated, so more prospective
331 studies are needed to confirm these findings. Second, because this study was a
332 single-center study with a relatively narrow group of participants, the findings
333 may not be well extrapolated to other populations. Finally, although we have
334 carefully adjusted for potential confounding factors, it is difficult to rule out
335 potential residual confounding.

## 336 Conclusion

337 Atherosclerosis is a chronic inflammatory disease of the walls of blood
338 vessels. In this study, novel inflammatory markers have good predictive
339 effects on carotid atherosclerosis, and NLPR has the highest predictive value.
340 NLPR can be used as a potential predictor of carotid atherosclerosis.

## 341 Funding

342 The study was supported by the National Natural Science Foundation of

343	China (Grant No.81571373, No.81601217, No.82001491), Natural Science

344 Foundation of Hubei Province of China (Grant No. 2017CFB627), Health
345 Commission	of	Hubei	Province	scientific	research	project	(Grant	No.
346 WJ2021M247) and Scientific Research Fund of Wuhan Union Hospital (Grant
347 No.2019).

## 348 Statements and Declarations

349 The authors declare that they have no known competing financial interests
350 or personal relationships that could have appeared to influence the work
351 reported in this paper.

## 352 Ethical statement

353 The ethical approval of the study is obtained from the Ethics Committee of Union
354 Hospital, Tongji Medical College, Huazhong University of Science and Technology at
355 Sep. 4th 2023 ([2023]ID:0611).

356

## 357 References

358 1.	Diseases GBD, Injuries C. Global burden of 369 diseases and injuries in 204
359 countries and territories, 1990-2019: a systematic analysis for the Global Burden of
360 Disease Study 2019. Lancet. 2020;396(10258):1204-22.
361 2.	Mensah GA, Roth GA, Fuster V. The Global Burden of Cardiovascular Diseases
362 and Risk Factors: 2020 and Beyond. Journal of the American College of Cardiology.
363 2019;74(20):2529-32.
364 3.	Roth GA, Mensah GA, Johnson CO, Addolorato G, Ammirati E, Baddour LM, et
365 al. Global Burden of Cardiovascular Diseases and Risk Factors, 1990-2019: Update
366 From the GBD 2019 Study. Journal of the American College of Cardiology.
367 2020;76(25):2982-3021.
368 4.	McGill HC, Jr., McMahan CA, Herderick EE, Malcom GT, Tracy RE, Strong JP.
369 Origin of atherosclerosis in childhood and adolescence. Am J Clin Nutr. 2000;72(5
370 Suppl):1307S-15S.
371 5.	Piepoli MF, Hoes AW, Agewall S, Albus C, Brotons C, Catapano AL, et al. 2016
372 European Guidelines on cardiovascular disease prevention in clinical practice: The
373 Sixth Joint Task Force of the European Society of Cardiology and Other Societies on
374 Cardiovascular Disease Prevention in Clinical Practice (constituted by representatives
375 of 10 societies and by invited experts)Developed with the special contribution of the
376 European Association for Cardiovascular Prevention & Rehabilitation (EACPR).
377 European heart journal. 2016;37(29):2315-81.
378 6.	Bos D, Arshi B, van den Bouwhuijsen QJA, Ikram MK, Selwaness M, Vernooij
379 MW, et al. Atherosclerotic Carotid Plaque Composition and Incident Stroke and
380 Coronary	Events.	Journal	of	the	American	College	of	Cardiology.
381 2021;77(11):1426-35.
382 7.	Nguyen MT, Fernando S, Schwarz N, Tan JT, Bursill CA, Psaltis PJ.
383 Inflammation as a Therapeutic Target in Atherosclerosis. Journal of clinical medicine.
384 2019;8(8).
385 8.	Chen Q, Che M, Shen W, Shao L, Yu H, Zhou J. Comparison of the Early
386 Warning Effects of Novel Inflammatory Markers SIRI, NLR, and LMR in the
387 Inhibition of Carotid Atherosclerosis by Testosterone in Middle-Aged and Elderly
388 Han Chinese Men in the Real World: A Small Sample Clinical Observational Study.
389 Am J Mens Health. 2023;17(3):15579883231171462.
390 9.	Geng L, Zuo L, Li H, Wang Y, Zhang Q, Ran J, et al. Association of
391 platelet-to-neutrophil ratios with 1-year outcome and mortality in patients with acute
392 ischemic stroke. Neurosci Lett. 2023;798:137016.
393 10. Guvenc O, Engin M. The role of neutrophil-lymphocyte platelet ratio in
394 predicting in-hospital mortality after acute Type A aortic dissection operations. Eur
395 Rev Med Pharmacol Sci. 2023;27(4):1534-9.
396 11. Karadeniz F, Karadeniz Y, Altuntaş E. Systemic immune-inflammation index, and
397 neutrophilto-lymphocyte and platelet-to-lymphocyte ratios can predict clinical
398 outcomes in patients with acute coronary syndrome. Cardiovascular journal of Africa.
399 2023;34:1-7.
400 12. Aydin C, Engin M. The Value of Inflammation Indexes in Predicting Patency of
401 Saphenous Vein Grafts in Patients With Coronary Artery Bypass Graft Surgery.
402 Cureus. 2021;13(7):e16646.
403 13. Song Y, Zhao Y, Shu Y, Zhang L, Cheng W, Wang L, et al. Combination model of
404 neutrophil to high-density lipoprotein ratio and system inflammation response index is
405 more valuable for predicting peripheral arterial disease in type 2 diabetic patients: A
406 cross-sectional study. Frontiers in endocrinology. 2023;14:1100453.
407 14. Kolaczkowska E, Kubes P. Neutrophil recruitment and function in health and
408 inflammation. Nat Rev Immunol. 2013;13(3):159-75.
409 15. Theofilis P, Oikonomou E, Tsioufis K, Tousoulis D. The Role of Macrophages in
410 Atherosclerosis: Pathophysiologic Mechanisms and Treatment Considerations. Int J
411 Mol Sci. 2023;24(11).
412 16. Nording HM, Seizer P, Langer HF. Platelets in inflammation and atherogenesis.
413 Front Immunol. 2015;6:98.
414 17. Wang Y, Wang R, Bai L, Liu Y, Liu L, He L, et al. The Leukocyte Subtype Counts
415 and Ratios Can Effectively Predict the Risk of Arterial Stiffness Assessed by
416 Cardio-Ankle Vascular Index: A Retrospective Study. Frontiers in cardiovascular
417 medicine. 2021;8:671885.
418 18. Albisinni S, Pretot D, Al Hajj Obeid W, Aoun F, Quackels T, Peltier A, et al. The
419 impact	of	neutrophil-to-lymphocyte,	platelet-to-lymphocyte	and
420 haemoglobin-to-platelet ratio on localised renal cell carcinoma oncologic outcomes.
421 Prog Urol. 2019;29(8-9):423-31.
422 19. Łochowski M, Łochowska B, Zawadzka I, Cieślik-Wolski B, Kozik D, Kozak J.
423 Prognostic	value	of	neutrophil-to-lymphocyte,	platelet-to-lymphocyte	and
424 lymphocyte-to-monocyte ratio ratios in patients operated on due to non-small cell
425 lung cancer. Journal of thoracic disease. 2019;11(8):3377-84.
426 20. Yang Z, Qiao Y, Wang D, Yan G, Tang C. Association Between Inflammatory
427 Biomarkers and Contrast-induced Acute Kidney Injury in ACS Patients Undergoing
428 Percutaneous	Coronary	Intervention:	A	Cross-sectional	Study.	Angiology.
429 2023:33197231185445.
430 21. Li Y, Zou Z, Zhang Y, Zhu B, Ning Y, Shen B, et al. Dynamics in perioperative
431 neutrophil-to-lymphocyte*platelet ratio as a predictor of early acute kidney injury
432 following cardiovascular surgery. Renal failure. 2021;43(1):1012-9.
433 22. Li S, Yu S, Qin J, Peng M, Qian J, Zhou P. Prognostic value of platelet
434 count-related ratios on admission in patients with pyogenic liver abscess. BMC Infect
435 Dis. 2022;22(1):636.
436 23. Chae YJ, Lee J, Park JH, Han DG, Ha E, Yi IK. Late Mortality Prediction of
437 Neutrophil-to-Lymphocyte and Platelet Ratio in Patients With Trauma Who
438 Underwent Emergency Surgery: A Retrospective Study. J Surg Res. 2021;267:755-61.
439 24. Fois AG, Paliogiannis P, Scano V, Cau S, Babudieri S, Perra R, et al. The
440 Systemic Inflammation Index on Admission Predicts In-Hospital Mortality in
441 COVID-19 Patients. Molecules. 2020;25(23).
442 25. Fan W, Wei C, Liu Y, Sun Q, Tian Y, Wang X, et al. The Prognostic Value of
443 Hematologic Inflammatory Markers in Patients With Acute Coronary Syndrome
444 Undergoing	Percutaneous	Coronary	Intervention.	Clinical	and	applied
445 thrombosis/hemostasis : official journal of the International Academy of Clinical and
446 Applied Thrombosis/Hemostasis. 2022;28:10760296221146183.

447

数字	[Click here to access/download;Figure;Fig 1.tif](https://www.editorialmanager.com/pone/download.aspx?id=34935220&guid=8e733fe2-e3c0-424f-8b0a-600e8c6a7fec&scheme=1)

数字	[Click here to access/download;Figure;Fig 3.tif](https://www.editorialmanager.com/pone/download.aspx?id=34935225&guid=f72bee84-5599-4f3f-89e3-d0243d96b61d&scheme=1)

Figure	[Click here to access/download;Figure;Fig 2.tif](https://www.editorialmanager.com/pone/download.aspx?id=34935504&guid=6486f21e-5ba3-4e69-8124-ad1e9b9c52a3&scheme=1)

Supporting Information

[Click here to access/download](https://www.editorialmanager.com/pone/download.aspx?id=35221641&guid=ab507b4e-eb00-4da8-afb3-d66d2f44845b&scheme=1)

# [Supporting Information](https://www.editorialmanager.com/pone/download.aspx?id=35221641&guid=ab507b4e-eb00-4da8-afb3-d66d2f44845b&scheme=1)

[Ethical review decision document-original letter.pdf](https://www.editorialmanager.com/pone/download.aspx?id=35221641&guid=ab507b4e-eb00-4da8-afb3-d66d2f44845b&scheme=1)

Supporting Information

[Click here to access/download](https://www.editorialmanager.com/pone/download.aspx?id=35221643&guid=8ec3bca5-5fd5-4147-b71e-f8e70d15e35a&scheme=1)

# [Supporting Information](https://www.editorialmanager.com/pone/download.aspx?id=35221643&guid=8ec3bca5-5fd5-4147-b71e-f8e70d15e35a&scheme=1)

[Ethical review decision document-English translated version.docx](https://www.editorialmanager.com/pone/download.aspx?id=35221643&guid=8ec3bca5-5fd5-4147-b71e-f8e70d15e35a&scheme=1)
